# Advanced Optical Detection through the Use of a Deformably Transferred Nanofilm

**DOI:** 10.3390/nano11030816

**Published:** 2021-03-23

**Authors:** Kossi Aniya Amedome Min-Dianey, Top Khac Le, Jeong Ryeol Choi, Phuong V. Pham

**Affiliations:** 1 Département de Physique, Faculté Des Sciences (FDS), Université de Lomé, Lomé 01BP1515, Togo; anyaratt20@yahoo.fr; 2 Department of Physics, Energy Harvest Storage Research Center, University of Ulsan, Ulsan 44610, Korea; lekhactop@gmail.com; 3 Department of Nanoengineering, Kyonggi University, Suwon 16227, Korea; 4 SKKU Advanced Institute of Nano Technology, Sungkyunkwan University, Suwon 440746, Korea

**Keywords:** nanofilm, deformable surface, smooth surface, IPA injection and N_2_ blowing, DI water injection and vdW interaction

## Abstract

Graphene has been extensively investigated in advanced photodetection devices for its broadband absorption, high carrier mobility, and mechanical flexibility. Due to graphene’s low optical absorptivity (2.3%), graphene-based photodetection research so far has focused on hybrid systems to increase photoabsorption. However, such hybrid systems require a complicated integration process and lead to reduced carrier mobility due to heterogeneous interfaces. Crumpled or deformed graphene has previously been reported in electronics and optoelectronics. However, a depth study on the influence of the morphology of nanofilms (e.g., graphite or graphene) related to light absorption in photodetection devices has not been demonstrated yet. Here, we present an interesting study in terms of the effect of the deformable surface and the smooth surface of a nanofilm transferred onto Si through two transfer strategies using isopropanol injection and nitrogen blowing (to form a deformable nanofilm surface) and deionized water injection and van der Waals interaction (to form a smooth nanofilm surface). As a result, optical detection in the case of the deformable nanofilm surface was enhanced significantly (~100%) compared with that of the smooth nanofilm surface in the visible laser wavelength (532 nm). In addition, evidence from the computational simulation also firmly affirms an advancement in the optical detection of deformed nanofilm-surface-based photodetection devices compatible with the experimental results.

## 1. Introduction

Graphene or monoatomic-layer graphite is a zero-bandgap honeycomb flat film formed by the sp^2^ hybridization of carbon atoms with different behaviors, e.g., high mobility, high transmittance, and novel thermal and electrical conductance [1,2,3,4,5,6,7,8,9,10,11,12,13,14,15,16,17,18,19,20,21,22,23,24,25,26,27,28,29,30]. In addition, it is well known as a promising sensing platform for detecting individual molecules leading to ultimate sensitivity for gas sensing [22,23], pressure sensing, [13,14,15,16,17,18,19,20,21,22,23,24], photodetection [17,18,19], biosensing [20], strain sensing [21], and temperature sensing [22]. Graphene films of a crumpled or rough shape are widely used, for instance, in electronics [23], energy storage [24,25], composites [26,27], and biomedicine [28].

Although it is known that the bendable degree of crumpling affects graphene’s properties and the performance of graphene-based devices and materials [25,27,29], the understanding of deformed graphene (or graphite) nanofilms on photodetection has not yet been adequately demonstrated. The crumpling of single-layer graphene has been reported on stretchable polymer substrates such as polydimethylsiloxane (PDMS) or very-high-bonding (VBH) tapes and Ecoflex by Zang et al. [30] and Kang et al. [31]. Zang et al. presented a strategy for controlling the crumpling and unfolding of large-area graphene placed on uniaxially relaxed PDMS or biaxially relaxed PDMS [30]. Through the control of prestrain relaxation, graphene film can be crumpled into tailored self-organized hierarchical structures. Moreover, this crumpled graphene–polymer laminate enables the design of large-area conductive coatings, superhydrophobic electrodes, or artificial muscle actuators [30]. In another report, Kang et al. revealed VHB- and Ecoflex-based stretchable photodetection devices based exclusively on crumpled graphene, which exhibited improved and strain-tunable photoresponsivity [31]. In addition, a computational calculation using a finite-element-method simulation (COMSOL) program was performed to prove graphene that crumpled at the edge sites of devices shows higher light adsorption than other regions of covered graphene [32]. This is great proof of the crumpled effect of graphene on visible and infrared photodetection. Although the reports above systematically investigated the crumpled effect of graphene and showed its contribution to light absorption enhancement, the crumpling effect of multilayer graphene or graphite created by the transfer process has not yet been established and requires further study.

In this study, we report the influence of the morphologies of 2D nanofilm materials on optical detection in terms of the visible wavelength under two shapes of transferred-nanofilm materials: (i) a deformable nanofilm surface formed by isopropanol (IPA) injection and N_2_ blowing, where N_2_ blowing makes the deformable nanofilm surface, and (ii) a smooth nanofilm surface formed by deionized (DI) water injection and van der Waals (vdW) interaction. Consequently, we achieved improved photoresponsivity by increasing the deformable density of the nanofilm surface compared with the smooth surface state.

## 2. Materials and Methods

Material Fabrication: The nanofilm was fabricated according to a previous report by Sone et al. [33] through the exfoliation of a highly oriented pyrolytic nanofilm crystal micro sheet.

Device Fabrication: The nanofilm was transferred onto a Si surface to cover the window of the photodetection device (Figure S1). Ethyl alcohol was then used to fill the gap between the nanofilm and Si and unfold the folds. The device was fabricated on a SiO_2_ layer (100 nm thick with n-doped Si (resistivity = ~10 Ω cm)). (i) Top metal electrode: The SiO_2_ layer was patterned by UV lithography. E-beam deposition and thermal evaporation processes were used to deposit the Ti/Au film as contact pads onto SiO_2_. The thicknesses of Ti/Au were 4 and 60 nm, respectively. (ii) Si window: After lifting off and cleaning, photolithography was used to pattern the windows with sizes of 5 × 5 mm^2^. SiO_2_ in the window was subsequently etched away by using a buffered oxide etchant, where the n-type Si was exposed for nanofilm/Si Schottky junction fabrication. (iii) Nanofilm transfer: To form a nanofilm/Si Schottky junction, the nanofilm film was first transferred to the top of the etched Si covering the window and metal pad. After a drying process at room temperature, ohmic contact between the nanofilm and Si was made. (iv) Patterning: The device was further patterned by UV lithography to etch the nanofilm outside the Ti/Au top electrode by O_2_ plasma. (v) Bottom metal electrode and wire bonding: (vi) After the nanofilm was patterned, GaIn paste was used to form an ohmic contact on the back side of the Si and Cu tape. (vii) Finally, Au wire bonding was used to connect the top electrodes, followed, finally, by packaging for the characterizations of the devices.

Characterization: Raman spectra (RM-1000 Invia, Renishaw plc., Wolton-under-Edge, Gloucestershire, UK) with an excitation energy of 2.41 eV (514 nm, Ar+ ion laser) were used for the characterization of the as-fabricated nanofilm. A field-emission scanning electron microscope (FE-SEM, Hitachi S-4700, Michigan Tech., Houghton, MI, USA) was used to observe the morphology for the two cases of the deformable nanofilm surface and the smooth nanofilm surface transferred onto Si. An atomic force microscope (AFM, Bruker corporation, Billerica, MA, USA) was used to measure the surface roughness for the two cases of the deformable nanofilm surface and the smooth nanofilm surface transferred onto Si. A transmission electron microscope (TEM, FEI Titan 80/300, FEI company, Hillsboro, OR, USA) was used to observe the surface morphology of the as-fabricated nanofilm. I–V curves were measured with the Agilent Semiconductor Analyzer B1500 using a lase with a visible wavelength of 532 nm for the two transferred-nanofilm/Si photodetection devices: deformable surface and smooth surface.

Computation: To confirm our experiments, a 3D finite-difference time-domain (FDTD) technique using Lumerical (Release 2017a, Ansys Ltd., Suite, Montreal, Canada) was utilized. A plane wave source with a polarization direction oriented along the *x*-direction was vertically irradiated on the structures along the *z*-direction (normal incidence). The incident wavelengths swept from 400 to 700 nm. The FDTD region had periodic boundary conditions on the sides (*x-* and *y*-directions) and absorbed perfectly matched layers in the direction of propagation to prevent boundary reflection. A graded mesh with “conformal variant 1” was used to achieve high accuracy simulation with full coverage of the structure. A profile monitor was used to record the reflected and transmitted fields through the structure once the convergence criterion was satisfied. This was followed by the absorption spectra A(λ), which was computed using the conservation law by A(λ) + R(λ) + T(λ) = 1, where λ is the wavelength, and R(λ) and T(λ) are reflectance and transmittance spectra, respectively. 

## 3. Results and Discussion

**Figure 1:**
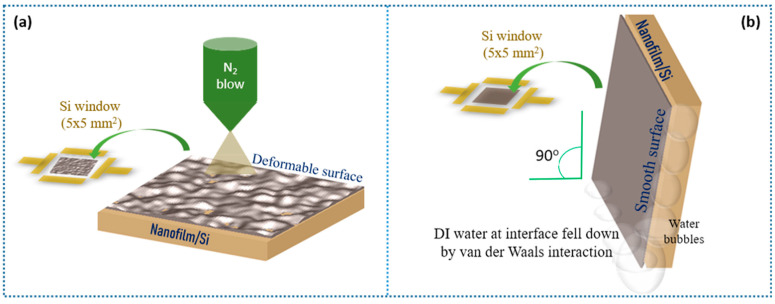
(**a**) Cross-sectional TEM image of the as-fabricated nanofilm. Two transfer methods of the nanofilm onto Si via (**a**) isopropanol (IPA) injection and N_2_ blowing (deformable surface) and (**b**) deionized (DI) water injection and van der Waals (vdW) interaction (smooth surface).

Figure S2 shows a cross-sectional TEM image of a nanofilm fabricated similarly to the previous report by Sone et al. [33]. Here, a nanofilm was created by stacked multiple layers, where the thickness of the nanofilm is ~12 nm, and each layer is ~0.34 nm. To analyze the multiple-layer phasing of this as-fabricated nanofilm, Raman spectra were captured (Figure S3). Figure 1a shows the formation of the deformable nanofilm on the Si window through the transfer step based on IPA injection and N_2_ blowing. In detail, the gap between the nanofilm and Si was easily removed by an injection tool that stored IPA solvent and slowly blew (N_2_) IPA bubbles at the interface with N_2_ to release it. Finally, it formed a deformable nanofilm surface, as shown in Figure 1a. On the contrary, Figure 1b describes a transfer method to form a smooth nanofilm surface through the injection of DI water. This nanofilm/Si was left in a vertically standing state for 5–10 min in the air in order to create the vdW interaction force that forms between the nanofilm and the macroscopic material (e.g., 3D Si substrate). This vdW interaction extremely attracts and causes adhesion at the interface between the nanofilm and Si to form a smooth nanofilm surface.

Figure S4 shows the morphologies of the deformable and smooth surfaces of the nanofilm after the nanofilm transfer steps shown in Figure 1. The deformable nanofilm surface was formed by folding, unfolding, tearing, point defects, multilayer, and the grain boundary, resulting in high roughness (Figure S4a) compared with the smooth nanofilm surface, which was made significantly less rough, with less folding and fewer point defects, owing to the vdW interaction (Figure S4b). As a result, the roughness established for the deformable nanofilm and the smooth nanofilm was 870.9 and 47.2 nm at that scale bar of 30 µm through AFM measurement, respectively (Figure S5).

**Figure 2:**
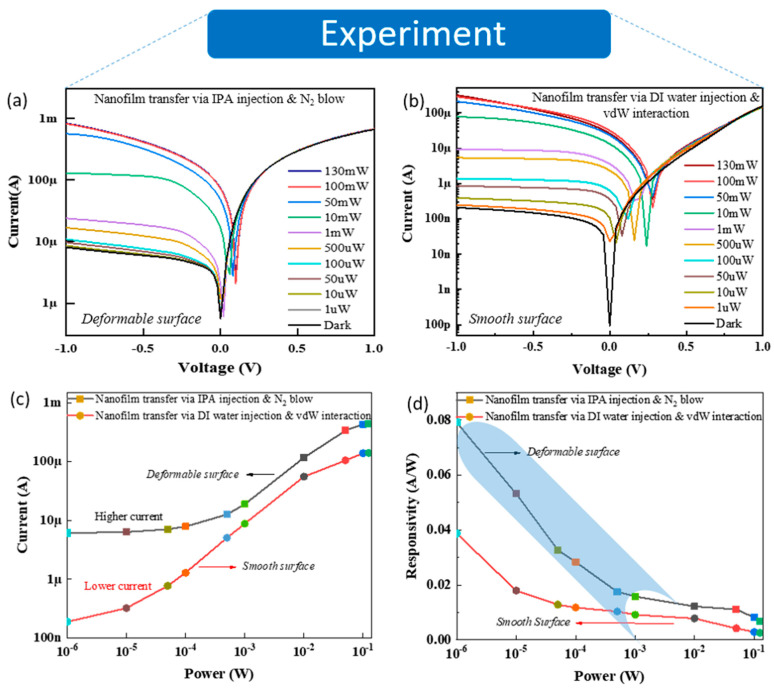
I–V curves of various laser powers measured on two different nanofilm-based photodetection devices using two transfer methods: (**a**) IPA injection and N_2_ blowing (deformable surface) and (**b**) DI water injection and vdW interaction (smooth surface). (**c**) Current and (**d**) responsivity of V = −1 V as the functions of different powers using a visible wavelength laser of the two transferred-nanofilm-based devices above.

**Figure 3:**
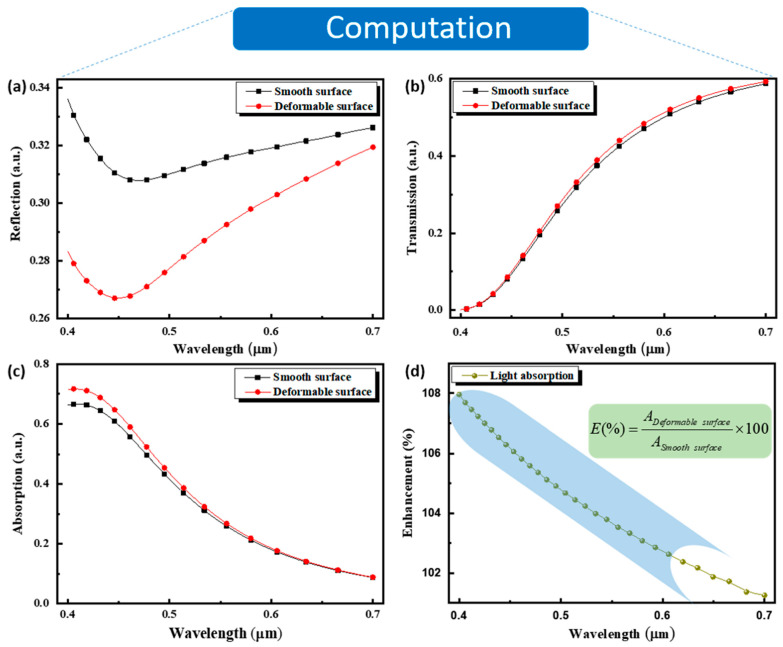
Lumerical finite-difference time-domain (FDTD) simulation for the (**a**) reflection spectra, (**b**) transmission spectra, (**c**) absorption spectra, and (**d**) light absorption enhancement of the photodetection devices based on the deformable and smooth nanofilm surfaces.

**Figure 4:**
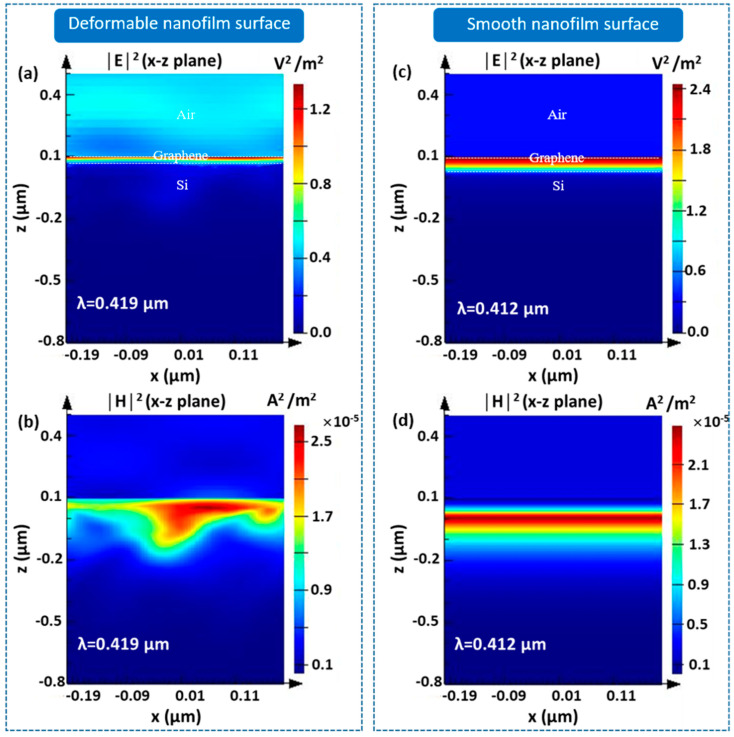
Simulated nearfield cross-sections at the resonance wavelength: electric field and magnetic field induced by (**a**,**b**) deformable and (**c**,**d**) smooth nanofilm surface structures, respectively.

To highlight the effect of the deformable nanofilm surface on light detection, the photodetection devices were fabricated on the SiO_2_ substrate, and the deformable and smooth nanofilms were transferred onto the etched Si window of the devices. Figure 2a,b reveals the dark current (I_dark_) and the photocurrent (I_light_) as functions of bias voltage (V), varying from –1 to 1 V, with exposure under a beam of 532 nm visible light and various incident power values (P_incident_) ranging from 1 µW to 130 mW. The more the laser power intensity is increased, the more the photocurrent at the reverse bias voltage (–1 to 0) increases. Here, the deformable nanofilm-based device showed higher dark current values, believed to be related to the fewer contacting points at the interface between the deformable nanofilm and Si. In addition, dark current values were obtained from multiple devices and showed similar results for the higher dark currents of the deformable nanofilm-based devices (Figure S6). Consequently, it led to a higher and clearer current increase in the deformable nanofilm compared with the smooth nanofilm, as shown in Figure 2c. This is a pioneering finding in this field that has not been investigated in any previous reports. In addition, to further clarify the mechanism of the higher dark current of the deformable nanofilm-based device, we investigated the computation method, which is presented in Figure 3 and Figure 4. In optoelectronics, photoresponsivity [R = (I_light_ − I_dark_)/P_incident_] is one of the key parameters to characterize the properties of optical detection. As seen in Figure 2d, the photoresponsivity values for the two cases above (Figure 2a,b) were converted at the reverse bias of −1 V as the function of various visible laser power intensities. As a result, all photoresponsivity values of the deformable surface were higher than that of the smooth surface. In particular, the highest light photoresponsivity was achieved at 0.08 A/W in the deformable surface, which was two times larger than the highest photoresponsivity of the smooth surface (0.04 A/W). This is an obvious experimental proof to show the crumple (or deformable) effect [30,31] of the nanofilm through the transfer process that is able to enhance light absorption. In addition, the values of noise-equivalent power (NEP) are given by $NEP=\frac{\sqrt{2e.I_{dark}}}{R.\sqrt{A}}$ (where R is responsivity, and A is the effective area (5 × 5 mm^2^)) of the deformable surface and the smooth surface, calculated as 2 × 10^−11^ and 4 × 10^−11^ W/(Hz)^1/2^, respectively.

To confirm our experiments above and reveal the enhancement mechanism in the visible spectra, both smooth and deformable surface structures were simulated using the 3D FDTD technique with Lumerical FDTD Solutions tools. Illustrations of the simulation model are shown as simulated nanofilm surfaces in Figure 1a,b. Figure 3 shows the simulated optical characteristics of the reflectance, transmittance, and absorptivity spectra through the smooth nanofilm and deformable nanofilm surface structures. This was used to investigate the effect exerted by the roughness of the deformable nanofilm structure compared to the smooth surface structure. As depicted in Figure 3a, the reflection decreases and attains a minimum peak at λ = 470 nm and λ = 455 nm for both the smooth and deformable surface structures, respectively. A gradual increase in reflection occurs along the visible spectrum, larger than the minimum peak wavelengths. A significant reflection loss from the deformable nanofilm surface compared with the smooth nanofilm surface is observed. Consequently, the deformable surface prevents reflection compared with that of the smooth surface. As the light is normally incident on the smooth nanofilm surface, a specular reflection occurs by reflecting the light in the direction from where it came. In the case of the deformable surface, the reflected light diverges from the specular reflection angle due to the roughness of the deformable surface. On the other hand, the low loss in reflection observed in the deformable surface structure can be attributed to multiple reflection events, which are expected to reduce the reflectance and increase the fraction of power absorbed. The deformable nanofilm surface plays the role of an antireflection structure, which is an alternative suitable method for absorption enhancement in a photodetection device, resulting in high device responsivity. The transmission in both smooth and deformable nanofilm surface structures is almost the same as that shown in Figure 3b. This suggests that the enhancement in light absorbed is governed by the internal reflection process. The absorption slightly increases in lower wavelengths of the visible spectrum in the deformable nanofilm surface compared with the smooth surface structure and remains the same from λ > 550 nm, as displayed in Figure 3c. This result is consistent with that reported by Bergström et al. [34]. This observation confirms that the deformable nanofilm surface pattern can enhance the collection efficiency of the absorption nanofilm layer, which is consistent with the prior reported study, where it was demonstrated that absorption depends on the correlation length and function, as well as the root mean square height of the rough surface, ensuring high absorptivity at higher frequencies [35]. A similar trend of absorption to that obtained in the responsivity in Figure 3d is seen. This similarity between the simulated absorption and measured responsivities suggests that the photocurrent enhancement is attributed to increased absorptivity, which is induced by roughness features in the nanofilm layer. The absorption enhancement E(%) is calculated according to E(%) = (A_Deformable surface_/A_Smooth surface_) × 100 in percentage, where A_Deformable surface_ and A_Smooth surface_ are the absorptivity in the deformable and smooth surface structures, respectively, and the result is depicted in Figure 3d. The enhancement is achieved by two orders of magnitude at lower wavelengths and can be strongly improved by controlling the roughness of the deformable nanofilm surface. As the photogenerated carrier density is directly proportional to the absorbed photons and the incident power, one can quantitatively enhance the photoelectric effect by a certain roughness threshold of the nanofilm layer in the visible spectrum near the ultraviolet domain. 

Figure 4 presents the simulated characteristics of the light-transmitted field for both the deformable nanofilm surface structure (Figure 4a,b) and the smooth nanofilm surface structure (Figure 4c,d) at resonance wavelengths of λ = 419 nm and λ = 412 nm, respectively. The electric and magnetic fields were computed at P-polarization and revealed a strong interaction in the nearfield distribution in the case of the deformable surface structure compared to that of the smooth surface, therefore confirming the enhancement in light absorption induced by the deformable features, as discussed in Figure 3d.

## 4. Conclusions

In summary, we demonstrated a simple nanofilm transfer method to enable the enhancement of photodetection. Consequently, increasing the areal deformable density of the nanofilm surface resulted in an order-of-magnitude enhancement of the optical extinction, which led to photoresponsivity enhancement (100%). This study will allow the realization of enhanced, strain-tunable, and wavelength-specific optoelectronics, as well as the enhancement and tuning of photodetection in future high-performance integrated optoelectronics. Although optical detection based on a deformably transferred nanofilm is enhanced, further improvement of the interface contact between the nanofilm and Si is required to obtain a more stable device structure with better contact. Our work could pave the way for other applications in electronics and optoelectronics in terms of ultraviolet-visible wavelength regions using other 2D thin films (e.g., graphene, hBN, transition metal dichalcogenides, or black phosphorous) integrated with this deformable transfer technique.

## Supplementary Materials

The following are available online at https://www.mdpi.com/2079-4991/11/3/816/s1, Figure S1: Fabrication process of nanofilm-based photodetection devices, Figure S2: Cross-sectional TEM image of the as-fabricated nanofilm, Figure S3: Raman spectra of the as-fabricated nanofilm, Figure S4: SEM images of the nanofilm transferred onto the Si window via (a) IPA injection and N_2_ blowing (deformable surface) and (b) DI water injection and vdW interaction (smooth surface), Figure S5: 3D AFM images of nanofilm transferred onto Si via (a) IPA injection and N_2_ blowing (deformable surface) and (b) DI water injection and vdW interaction (smooth surface), Figure S6: Dark currents measured on various nanofilm-based photodetection devices for two transfer methods via (a) IPA injection and N_2_ blowing (deformable surface) and (b) DI water injection and vdW interaction (smooth surface).
