# Supplementary material for: Advanced Optical Detection through the Use of a Deformably Transferred Nanofilm
Source: Nanomaterials (Basel). 2021 Mar 23;11(3):816. doi: 10.3390/nano11030816 (PMC8004814; doi:10.3390/nano11030816)
Supplement: Supplementary file 1 [file nanomaterials-11-00816-s001.pdf]

Supporting Information

Advanced Optical Detection through the Use of a Deformably Transferred Nanofilm

Kossi Aniya Amedome Min-Dianey [1], Top Khac Le [2], Jeong Ryeol Choi [3,*] Phuong V. Pham [4,*]

[1] Département de Physique, Faculté Des Sciences (FDS), Université de Lomé, Lomé 01BP1515, Togo; anyaratt20@yahoo.fr
[2] Department of Physics, Energy Harvest Storage Research Center, University of Ulsan, Ulsan 44610, Korea; lekhactop@gmail.com
[3] Department of Nanoengineering, Kyonggi University, Suwon 16227, Korea
[4] SKKU Advanced Institute of Nano Technology, Sungkyunkwan University, Suwon 440746, Korea
* Correspondence: jrchoi@kyonggi.ac.kr (J.R.C.); pvphuong@skku.edu (P.V.P.)

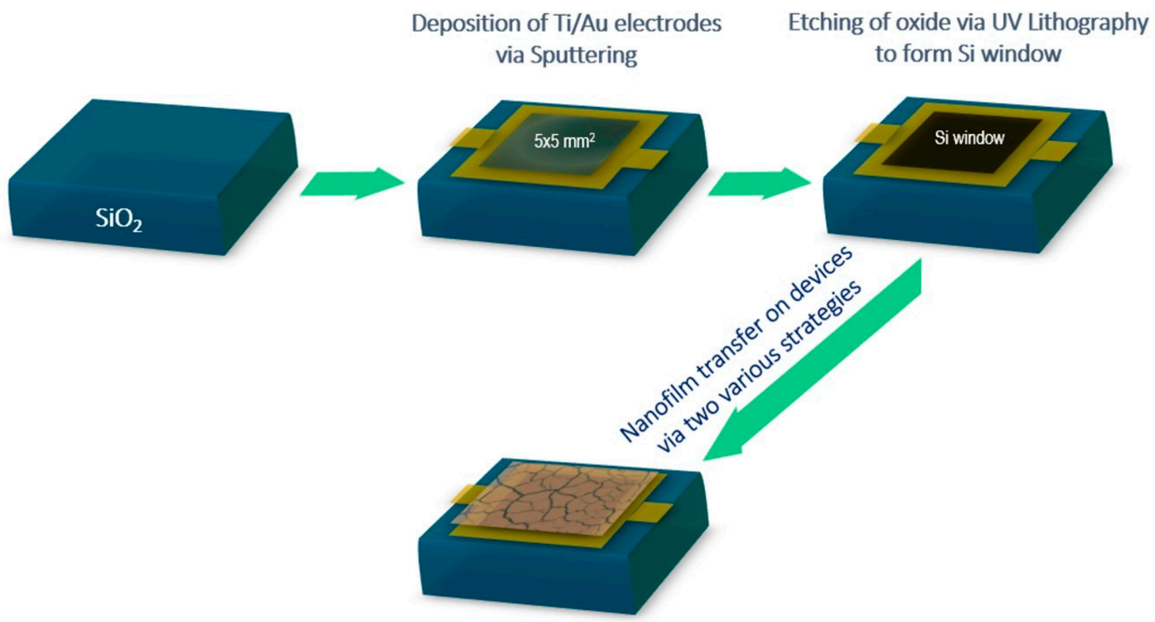

Figure S1. Fabrication process of nanofilm-based photodetection devices.

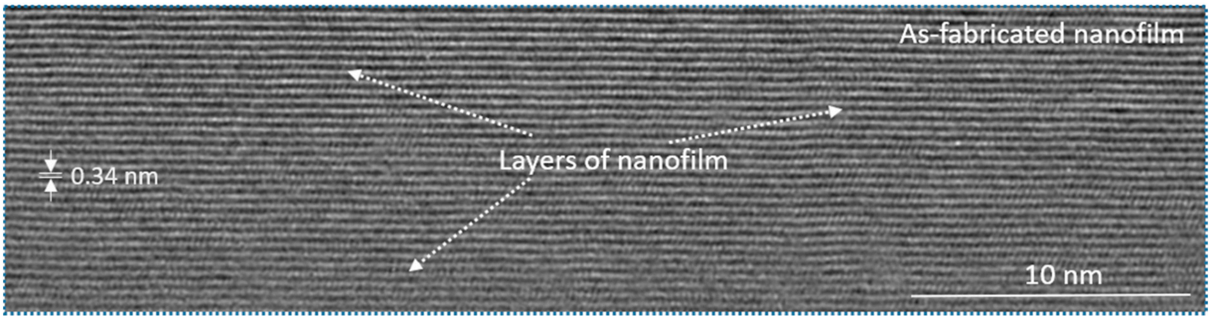

Figure S2. Cross-sectional TEM image of as-fabricated nanofilm.

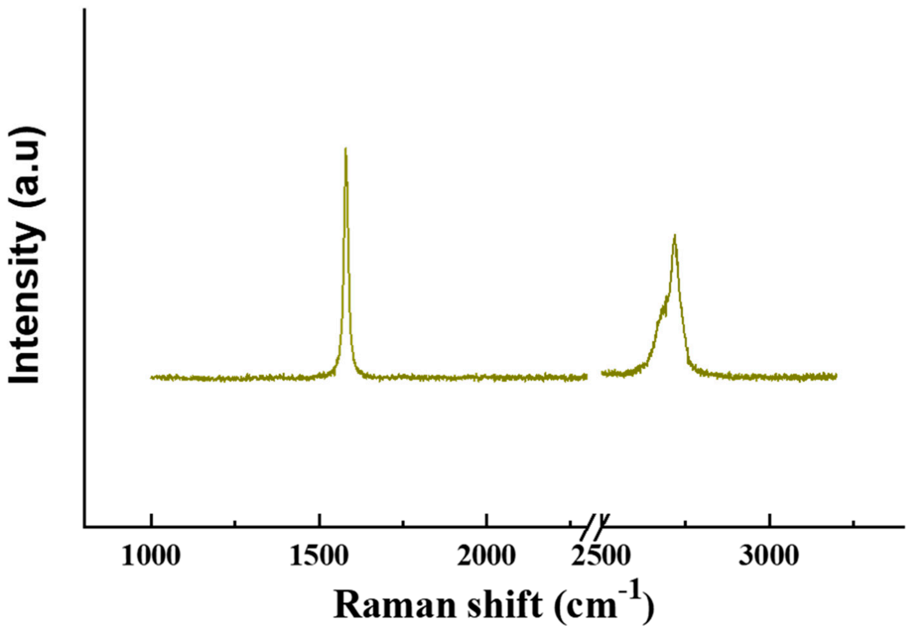

Figure S3. Raman spectra of as-fabricated nanofilm.

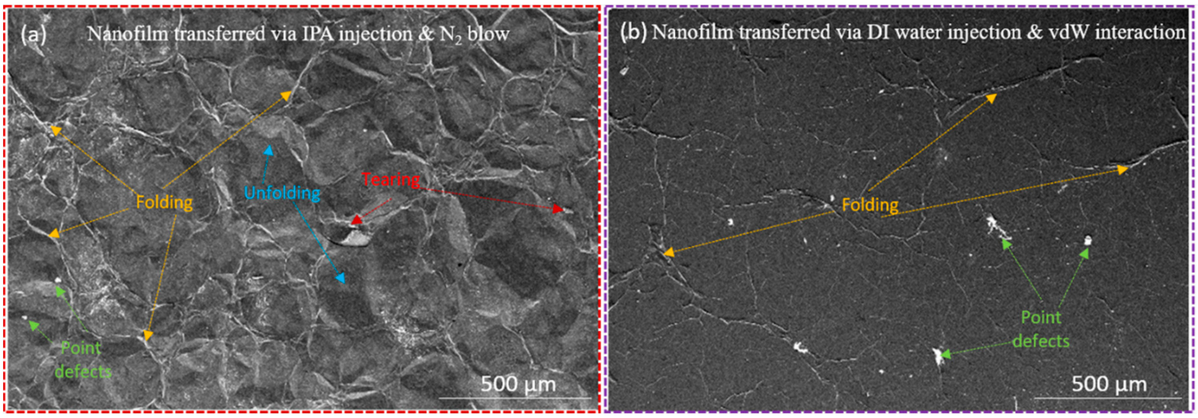

Figure S4. SEM images of nanofilm transferred on Si window via (**a**) IPA injection & N_2 blow (deformable surface) and (**b**) DI water injection & vdW interaction (smooth surface).

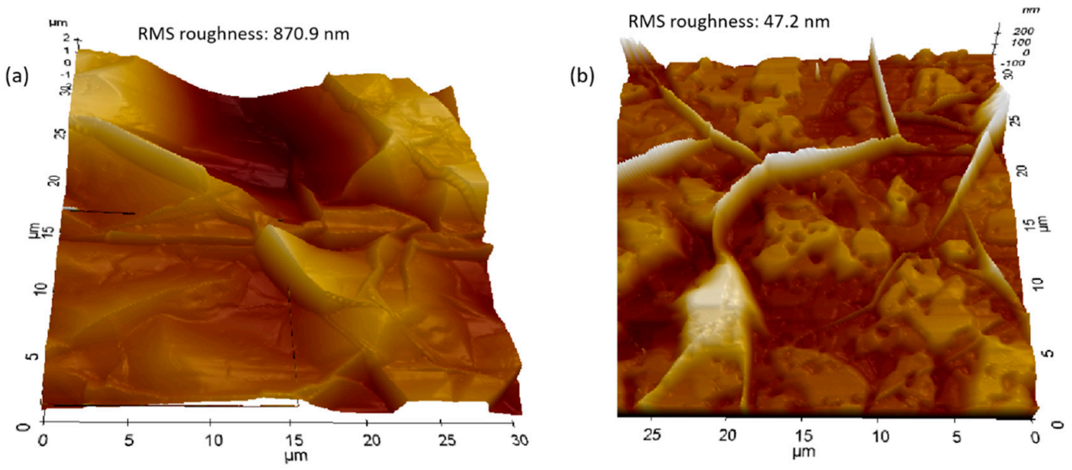

Figure S5. 3D AFM images of nanofilm-transferred on Si via (**a**) IPA injection & N_2 blow (deformable surface) and (**b**) DI water injection & vdW interaction (smooth surface).

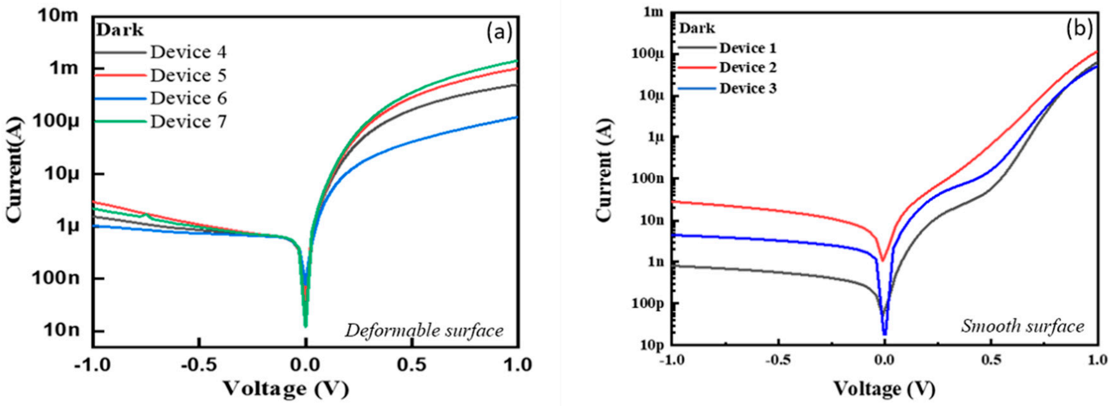

Figure S6. Dark currents measured on various nanofilm-based photodetection devices for two transfer methods via (**a**) IPA injection & N_2 blow (deformable surface) and (**b**) DI water injection & vdW interaction (smooth surface).
